# Factors associated with attitudes and beliefs of elders with acute low back pain: data from the study Back Complaints in the Elders (BACE)

**DOI:** 10.1590/bjpt-rbf.2014.0188

**Published:** 2016-09-22

**Authors:** Luiza F. Teixeira, Leani S. M. Pereira, Silvia L. A. Silva, João M. D. Dias, Rosângela C. Dias

**Affiliations:** 1 Programa de Pós-graduação em Ciências da Reabilitação, Escola de Educação Física, Fisioterapia e Terapia Ocupacional, Universidade Federal de Minas Gerais (UFMG), Belo Horizonte, MG, Brazil; 2 Departamento de Fisioterapia, Universidade do Vale do Sapucaí (UNIVAS), Pouso Alegre, MG, Brazil; 3 Departamento de Fisioterapia, Escola de Educação Física, Fisioterapia e Terapia Ocupacional, UFMG, Belo Horizonte, MG, Brazil; 4 Departamento de Fisioterapia, Universidade Federal de Alfenas (UNIFAL), Alfenas, MG, Brazil

**Keywords:** elderly, low back pain, psychosocial factors, rehabilitation

## Abstract

**Background:**

The attitudes and beliefs that older people have about acute low back pain (LBP) may influence the coping mechanisms and the adoption of treatment strategies in this population.

**Objective:**

The aim of this study was to identify the factors associated with the attitudes and beliefs of elderly patients with acute low back pain using the Back Beliefs Questionnaire.

**Method:**

This is a cross-sectional study with a subsample of the study “Back Complaints in the Elders” (BACE), composed of 532 older Brazilians of both genders with acute LBP. We investigated sociodemographic and clinical aspects, self-perceived health, psychosocial and emotional state, falls, and functional capacity. Multiple regression models were constructed to measure possible associations.

**Results:**

The percentage of female participants was 85.7% and the mean age was 69.04 (SD=6.2). Disability, symptoms of depression, and expectation of return to activities were independently associated with attitudes and beliefs concerning LBP.

**Conclusion:**

Screening of psychosocial factors is essential to the prevention of persistent and recurrent LBP. Early signs of these factors can help identify symptoms and behaviors for effective interventions.

## BULLET POINTS

• Negative attitudes and beliefs concerning low back pain are associated with disability.
• Symptoms of depression and expectation of return to activities are associated with attitudes and beliefs concerning LBP and its consequences.
• Screening of psychosocial factors can identify disability in the elderly.

## Introduction

Low back pain (LBP) is a common musculoskeletal condition with a worldwide prevalence of 11.9%^1^. In community-dwelling elders, the prevalence of LBP ranges from 13 to 49%^2^, however most studies have been aimed at young and middle-aged populations. Consequently, LBP in the elderly poses a challenge to the implementation of public policies^3^.

“Yellow flags”, in the context of LBP, are psychosocial factors that increase the risk of transition from acute to chronic LBP and the development of future disability^4^. Among the seven known yellow flags, the following factors should be identified^4^ ^,^ ^5^: inadequate attitudes and beliefs, inadequate behavior, problems related to workers’ compensation and issues in the workplace, issues involving diagnosis and treatment, emotional problems, and family issues^4^ ^,^ ^5^. There is an emphasis on the role of attitudes and beliefs as a factor associated with the chronification of LBP, resulting in decreased daily activity, disability, and social isolation^5^.

The negative attitudes and beliefs of patients or health professionals can limit the patient’s ability to cope with painful symptoms, and adopting passive treatment strategies may present a high risk of persistence of symptoms and disability. On the other hand, positive beliefs and attitudes in relation to LBP and behaviors that prevent kinesiophobia, for example, could be important factors in the prevention of disability in elders^6^ ^-^ ^8^. Both in research and in clinical practice, one of the main instruments used, is the Back Beliefs Questionnaire, which measures the individual’s attitudes and beliefs concerning the inevitable consequences of future life with LBP^9^.

With age, there is an increase in serious and disabling events of LBP that interfere with daily activities and restrict physical and social function. Therefore, greater attention should be given to LBP conditions in the elderly^7^ ^,^ ^10^. The aging process can contribute to an increase in negative attitudes and beliefs that lead elders to tolerate pain by attributing it to aging instead of seeking the help of health professionals and services. These negative attitudes can also lead health professionals to withdrawal from providing care to the elderly individual^3^ ^,^ ^10^. However, it is not known which factors have been associated with attitudes and beliefs in this specific elderly population and in acute LBP. Previous studies^8^ ^,^ ^11^ ^,^ ^12^ found only an association between psychosocial factors and disability, but the literature still has not found evidence of factors associated with the outcome “attitudes and beliefs concerning LBP” in this approach. It was hypothesized that older people with acute LBP who had negative attitudes and beliefs would present worse self-rated health, worse psychosocial and emotional status, greater incidence of falls, and greater functional disability compared to those who had positive beliefs and attitudes. Therefore, the objective of this study was to identify factors associated with attitudes and beliefs in elderly patients with acute LBP.

## Method

### Design and participants

The Back Complaints in the Elders (BACE) consortium is a prospective epidemiological study whose protocol has already been published^10^. This cross-sectional study included a sample of 532 individuals (age: 60 and over) with a new episode of LBP, defined as pain or discomfort between the costal margins and the inferior gluteal fold with or without referred pain to the lower limbs^13^. An episode was defined as new if the person had not required health care for LBP during the six months prior to the data collection^10^. The elders had to present with a worsening of symptoms, which was defined as an episode of acute pain within the six weeks prior to the recruitment period. Those who fit these criteria were invited to participate.

For the BACE-Brazil (BACE-B) study, the elders were recruited by convenience and advised by doctors and health professionals to contact our team when they presented with LBP. Next, they underwent a screening to determine whether they fit the criteria for inclusion in the study. Individuals were excluded if they presented with any serious motor, hearing, or visual disabilities that might prevent them from being evaluated or if they had cognitive impairment identified by the Mini-Mental State Examination, considering the cutoff points according to educational level: 13 points for illiterates, 18 points for those with up to 8 years of schooling, and 26 points for those with more than 8 years of schooling^14^. The BACE-B study was approved by the Research Ethics Committee of the Universidade Federal de Minas Gerais (UFMG), Belo Horizonte, MG, Brazil (approval number 0100.0.203.000-11). All participants signed an Informed Consent Form (ICF).

### Instruments

#### Sociodemographic and clinical characterization

The following demographic data were collected: gender (male/female), age, education (years of schooling), and current employment (yes/no). The mean age and education were calculated.

The intensity of current pain and pain in the previous week was measured using the Numerical Pain Scale (NPS) with scores ranging from 0 (no pain) to 10 (worst possible pain)^15^. The mean intensities of current pain and pain in the last week were calculated. The frequency of pain in the lower back, buttocks, or legs was categorized as follows: less than once a week and at least once a week; every day for at least a few minutes and every day for most of the day; or all of the time. Regarding treatment, the participants were asked to answer “yes” or “no” regarding whether they had (a) seen a health professional in the past six weeks for their LBP, (b) taken any medication for LBP in the past three months, or (c) received any treatment for pain (i.e. use of orthosis, back brace, or other therapies for pain).

#### Dependent variable

The Back Beliefs Questionnaire (BBQ)^9^ ^,^ ^16^ measured the individual’s attitudes and beliefs about the inevitable consequences of future life with LBP. It consisted of nine statements, along with five questions used as distractors, totaling 14 items. The participant reported the level of agreement on a 5-point Likert type scale (ranging from 1 - strongly disagree to 5 - strongly agree). The score is calculated by reversing the sum of the scores of the nine statements. The total score ranged from 9 to 45 points. The lower the score was, the greater the negative attitudes and beliefs concerning LBP. The BBQ had good validity, Cronbach's alpha of 0.70-0.75, and test-retest reliability of 0.87^9^. The BBQ score was computed continuously.

### Independent variables

#### Self-perception of health

##### Global perceived effect

The elders expressed their personal and subjective opinion comparing the initial symptoms of LBP to their condition at the time of the assessment^17^. The multiple-choice answers ranged from “fully recovered” to “worst possible pain” and were categorized as: fully recovered and greatly improved; improved slightly, remains the same, and a little worse; or much worse and worse than ever^17^.

##### Expectation of recovery and return to activities in three months

This outcome was investigated using two semi-structured questions: “How do you think your back pain will be in three months?” and “What is your expectation of returning to activities in three months?” The possible answers for the first question ranged from “totally pain free” to “worst possible pain” and were categorized as: totally pain free and considerable improvement; still the same; or much worse and worse than ever. For the second question, the answers ranged from “full return” to “no return at all” and were categorized as follows: full return and partial return; same as now; or less than before and no return at all.

### Psychosocial and emotional state

Symptoms of depression were assessed using the Center for Epidemiological Studies - Depression Scale (CES-D)^18^, which had satisfactory levels of internal validity (Cronbach alpha = 0.86), sensitivity (74.6%), and specificity (73.6%) and cutoff point >11, indicating worse depression^18^. The CES-D was computed continuously.

Self-efficacy for falls was evaluated using the Brazilian version of the Falls Efficacy Scale - International (FES-I-Brazil)^19^ in which individuals are asked to rate, from 1 to 4, their concerns about the possibility of falling while performing 16 daily activities. The total score could range from 16 (no concern) to 64 (extreme concern). The FES-I Brazil had adequate internal consistency (Cronbach alpha=0.93) and reliability (ICC=0.84 and 0.91, intra and interrater, respectively)^19^ and was computed continuously.

### Occurrence of falls

The participants were asked to provide the number of falls they had in the last six weeks, which corresponded to the period of LBP. Falls were computed continuously (total number of falls) and categorically (none, one, and two or more falls).

### Disability

Disability was assessed using the Roland Morris Disability Questionnaire (RMDQ)^20^, which measured LBP-related disability in 24 situations involving activities of daily living. Each question was given a score of 0 (no difficulty) or 1 (difficulty). The total score ranges from 0 to 24, with scores ≥14 points indicating greater disability. The test-retest reliability was 0.91 for same-day administration, ICC was 0.93 for 1 to 14 days and 0.86 for 3 to 6 weeks, and internal consistency was good (Cronbach’s alpha = 0.83)^20^. Disability was computed continuously.

### Functional mobility

Functional mobility was measured by the Timed Up and Go (TUG) test^21^, which had excellent interrater (ICC=0.98) and intra-rater reliability (ICC=0.99). During the test, the participants were instructed to rise from an armchair of standard height (45 cm) without help and with their arms crossed over their chest. On the word “Go”, the participant had to walk at a safe pace to a line on the floor 3 meters away, turn, return to the chair, and sit without hand support. The test was timed (seconds) and repeated twice with one minute of rest between repetitions. The mean of the times was calculated^21^. Bohannon^22^ suggested the following cutoffs according to age: 8.1 (7.1 to 9.0) seconds for 60 to 69 years old, 9.2 (8.2 to 10.2) seconds for 70 to 79 years old, and 11.3 (10.0 to 12.7) seconds for 80 to 99 years old^22^. The average time spent on the TUG was calculated.

### Gait speed

Normal gait speed (GS) was measured with a digital stopwatch while each participant walked along a level 4.6-meter path, with 2 extra meters for acceleration and 2 for deceleration, according to Fried et al.^23^. For the test, the participants wore normal footwear to walk at self-selected speed, which was measured in distance/time (m/s). The use of walking aids was allowed, and the mean GS was calculated.

### Statistical analysis

For sample characterization, descriptive analysis was conducted using simple frequency (percentages) for categorical variables and measures of central tendency (mean) and dispersion (standard deviation) for continuous and discrete variables. The Kolmogorov-Smirnov test verified the hypothesis of normal distribution of the data for all independent variables and for the BBQ.

Multiple linear regression analysis was used to identify the variables associated with the outcome attitudes and beliefs concerning LBP. To define the variables that would be used in the multivariate analysis, a Pearson correlation analysis was conducted between the continuous variables and the BBQ score, and an independent t test and one-way ANOVA between the categorical variables and the total score for the same questionnaire. Previous univariate analysis allowed only variables that might be associated with the outcome to be included in the multivariate analysis. Variables with a *p* value less than 0.20 were selected for analysis using the stepwise regression, which determined the addition and maintenance of the variables in the final models. Collinearity was verified using Pearson’s correlation analysis between continuous independent variables and Spearman’s correlation coefficient between the categorical variables and between the categorical and continuous variables. There was no multicollinearity preventing the construction of the three final models, with gradual addition of the variables as they were more associated with the dependent variable. Among all variables, the score in the depression scale was considered a control variable for addition to the model because of its influence on the attitudes and beliefs assessed by the BBQ. The variables with *p*<0.05 were considered and retained in the final models. The statistical software Statistical Package for Social Sciences (SPSS) version 18.0 was used for the statistical analysis.

## Results

**Figure 1:**
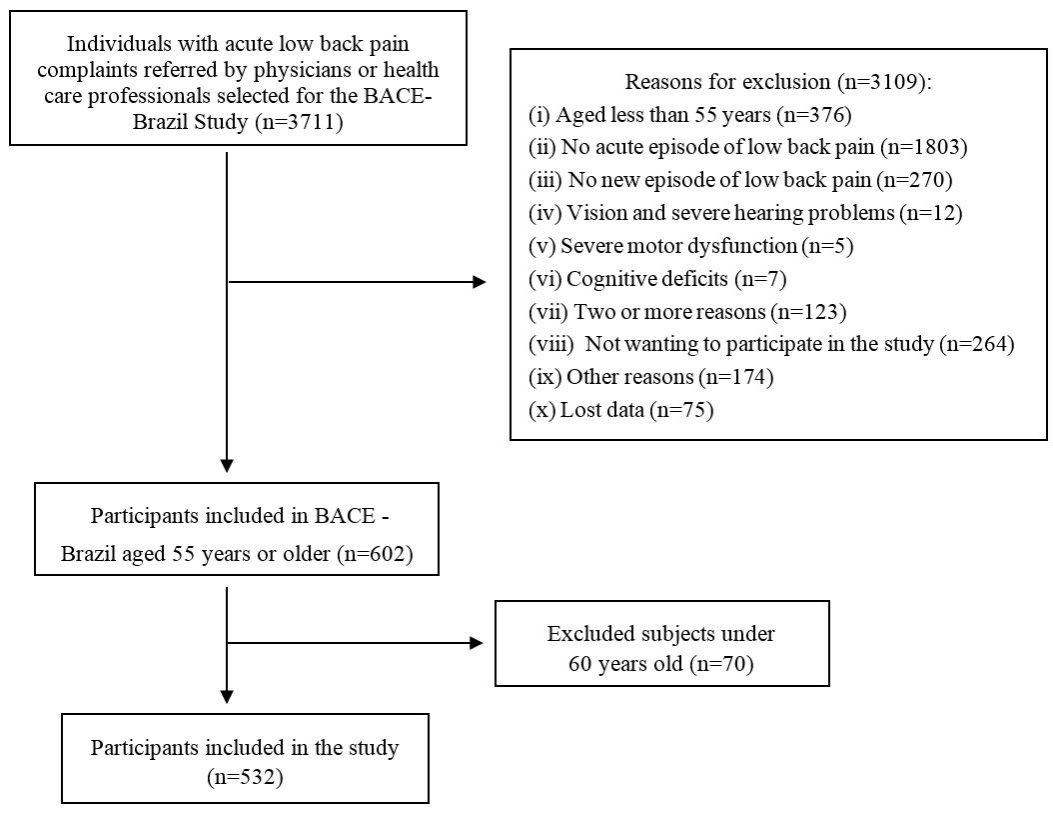
Flow chart of the study.

The flow diagram of the study is shown in Figure 1. The characterization of the 532 elders who participated in this study from September 2011 to September 2015 is presented in Tables 1 and 2 and the *missing values* in tables were due to lack of understanding of the questions by participants.

**Table 1:** Socio-demographic and clinical characteristics and occurrence of falls among elderly acute low back pain participants.

**Variable**	**n= 532**
**Sociodemographic data**	
**Woman**; n (%)	456 (85.7)
**Age (years);** mean (SD)	69.0 (6.2)
**Education (years)**; mean (SD)^*^	7.3 (4.8)
**Work (paid)**; n (%)	84 (15.8)
**Clinical data**	
**Intensity of current pain (NPS)**; mean (SD)	4.7 (3.0)
**Pain intensity in the last week (NPS)**; mean (SD)	7.1 (2.6)
**Pain frequency**; n (%)	
At least once a week	179 (33.6)
Every day	255 (47.9)
During all the time	98 (18.4)
**Consultation with healthcare**; n (%)	157 (29.5)
**Treatment;** n (%)	
LBP medication in the last 3 months	395 (74.2)
Use of orthosis (brace) or therapies for pain	78 (14.7)
**Falls**; mean (SD)	
**Number of falls**; n (%)^*^	
None	396 (74.4)
One fall	70 (13.2)
Two or more falls	66 (12.4)

LBP: Low Back Pain; NPS: Numerical Pain Scale; SD: Standard Deviation; n: number.

* *Missing values:* Education: n=5; **N**umber of falls and falls: n=7.

**Table 2:** Sample characterization of psychosocial, emotional, functional and self-perceived health variables among elderly, acute low back pain participants.

**Variable**	**n= 532**
**Psychosocial and emotional state**	
**Back Beliefs Questionnaire (BBQ)**; mean (SD)^*^	23.7 (6.6)
**Center for Epidemiologic Studies Depression (CES-D)**; mean (SD)^*^	18.2 (11.7)
**Falls Efficacy Scale International - Brazil (FES-I-Brazil)**; mean (SD)^*^	31.2 (9.2)
**Functional capacity**	
Roland Morris Disability Questionnaire (RMDQ); mean (SD)*	13.6 (5.8)
**Timed Up and Go(s)**; mean (SD)^*^	11.3 (3.9)
**Gait Speed (GS) (m/s)**; mean(SD)	1.0 (0.2)
**Self-perception health**	
**Perception of the overall effect**; n (%)	
Improvement	108 (20.3)
Remains the same	318 (59.8)
Worsens	106 (19.9)
**Expectation of recovery in 3 months**; n (%)	
Improvement	284 (53.4)
Still the same	172 (32.3)
Worsens	76 (14.3)
**Expectation of return to activities in 3 months (ERA 3m)**; n (%)^*^	
Return	476 (89.5)
Still the same	54 (10.2)
No return	0 (0.0)

LBP: Low Back Pain; SD: Standard Deviation; n: number; m/s: meters/second; s: second.

* *Missing values:* CES-D: n= 3; FES-I-Brazil: n=18; BBQ: n=1; RMDQ: n=35; GS: n=4; TUG: n=6; ERA 3m: n=2.

In the univariate analysis, the following variables presented *p*<0.20 in the correlation or difference tests between groups: symptoms of depression, self-efficacy for falls, disability, functional mobility, gait speed, and expected return to activities (Table 3). The variables that were significant in the univariate analysis were added as independent variables in the regression analysis, with depression being the control variable. The variables associated with attitudes and beliefs were disability, symptoms of depression, and expectation of return to activities, while the other variables (self-efficacy for falls, functional mobility, and gait speed) showed no association with attitudes and beliefs within the model and were excluded from the final model (Table 4).

**Table 3:** Univariate analysis of predictors of attitudes and beliefs about back pain for continuous and categorical variables among elderly, acute low back pain participants.

	CES-D	FES-I-Brazil	Falls	RMDQ	TUG	GS	
BBQ	n=528	n=513	n=524	n=496	n=525	n=527	
r	–0.331	–0.227	–0.052	–0.369	–0.085	0.117	
p	<0.001‡	<0.001‡	0.231	<0.001‡	0.051‡	0.007‡	
BBQ	Number of falls			Expectation of return to activities			
	Yes	No	p	Return	The same	No return	p
				(n=289)	(n=187)	(n=53)	
(mean	22.6	23.9	0.096†	24.3	23.8	19.5	<0.001^*^
and SD)	(6.6)	(6.6)		(7.0)	(5.7)	(6.3)	<0.001^**^
							0.652^***^

BBQ: Back Beliefs Questionnaire; CES-D: Center for Epidemiological Studies - Depression; FES-I-Brazil: Falls Efficacy Scale International-Brazil; RMDQ: Roland Morris Disability Questionnaire; TUG: Timed Up and Go; GS: Gait speed.

‡ p<0.20 (inputs in the final model).

r: Pearson's correlation analysis.

† Independent t test; one way ANOVA (post-hoc Tukey).

* p-value between groups “return” and “no return”;

** “the same” and “no return”;

*** “return” and “the same”;

SD: Standard Deviation Significant p<0.05.

**Table 4:** Multiple regression analysis of predictors of attitudes and beliefs about LBP among elderly, acute low back pain participants.

	Model 1	Model 2	Model 3
Constant (β)	29.39^*^	29.91^*^	31.55^*^
p	(<0.001)	(<0.001)	(<0.001)
IC95%	–0.520 to –0.331	–0.426 to –0.211	–0.427 to –0.214
RMDQ (β)	–0.414^*^	–0.306^*^	–0.310
p	(<0.001)	0.057	(<0.001)
IC95%	–0.520 to –0.331	–0.426 to –0.211	–0.427 to –0.214
CES-D (β)		–0.110^*^	–0.103^*^
p		(<0.001)	(>0.001)
IC95%		–0.161 to –0.054	–0.153 to –0.046
Expectation of return to activities (β)			–1.124^*^
p			(0.003)
IC95%			–2.064 to –0.423
R^2^	0.129	0.156	0.168

* Significant p<0.05.

Enters the regression model: FES-I-Brazil (Falls Efficacy Scale International-Brazil); Number of falls, RMDQ (Roland Morris

Disability Questionnaire); TUG (Timed Up and Go); Control: CES-D (Center for Epidemiologic Studies Depression); R^2^:

Magnitude of the correlation; LBP: Low Back Pain.

In model 1, disability was associated with attitudes and beliefs and with worse attitudes and beliefs concerning LBP. Disability explained 12.9% of the variation in the BBQ scores. In model 2, symptoms of depression were used as a confounding variable and the variability of disability decreased, however the model’s power of explanation increased only slightly (15.6%). In model 3, expectation of return to activities was not a confounding variable and helped to explain the variation in attitudes and beliefs concerning LBP by 16.8% (Table 4).

## Discussion

The association between attitudes and beliefs in elderly patients with acute exacerbated episode of LBP was investigated. The results showed that disability, symptoms of depression, and expectation of return to activities were associated with attitudes and beliefs concerning LBP.

The current study found an association between negative attitudes and beliefs (considered a psychosocial factor) and disability in elders. Some studies have identified the influence^8^ ^,^ ^11^ or lack of influence^12^ ^,^ ^24^ of psychosocial factors on disability in elderly patients with LBP, as well as other aspects such as sample and cultural profiles^8^ ^,^ ^11^ ^,^ ^12^ ^,^ ^24^.

In a study conducted in Australia with 542 community-dwelling women aged 24 to 80, negative attitudes and beliefs were identified in individuals with high intensities of pain and disability^8^. In contrast, the present study found no association between attitudes and beliefs and intensity of acute LBP, which could be explained by that study’s use of a non-specific sample of elderly patients and selection of chronic LBP^8^. Corroborating our study, a cross-sectional study with a sample of elderly Americans aged 79.74 (7.77) years showed an association between attitudes and beliefs and disability. The score of the Fear Avoidance Beliefs Questionnaire - Physical Activity subscale (FABQ-PA) explained 3 to 6% of the variance in disability related to LBP measured by the Quebec Back Pain Disability Scale^11^.

The studies of Kovacs et al.^12^ ^,^ ^24^ found no association between psychosocial factors and disability and differed from the present study in some aspects related to the sample and measuring instruments of psychosocial outcomes and LBP conditions. Kovacs et al.^24^ found no association between kinesiophobia and disability in elderly Spaniards with LBP, but there was an association between pain and disability. Unlike the present study, that study included a sample of institutionalized elders aged 80.4 (6.1) years and the duration of LBP was categorized as acute (<14 days), subacute (between 14 and 89 days), and chronic (≥90 days). The same research group^12^ conducted another study with community elders and found an influence of kinesiophobia and catastrophizing on LBP - related disability. In addition, they found moderate correlation of kinesiophobia (*r*=0.41) and catastrophizing (*r*=0.42) and no correlation with the intensity of LBP. In the regression model, there was a weak and clinically irrelevant association between kinesiophobia and disability (R=0.28; 95% CI 0.18 to 0.38) and catastrophizing (R=0.19; 95% CI 0.09 to 0.30)^12^. The psychosocial outcomes differ from the present study, as do the age used (65 or more) and LBP characterized as acute (<90 days) and chronic (≥90 days)^12^.

Attitudes and beliefs about LBP were associated with symptoms of depression in this study. According to Wand et al.^25^, pain-related disability impairs aspects of daily life and causes psychological distress. As noted in this study, older adults with complaints of musculoskeletal pain and symptoms of depression were less willing to perform daily activities and follow recommended directions and treatments^25^.

Some aspects, such as kinesiophobia and social isolation, could reduce self-efficacy and increase the chance of developing symptoms of depression and disability^25^. Corroborating the present study, Misterska et al.^26^ found that coping strategies for LBP were related to beliefs about the management of pain, depression, and disability, and in another study^27^, associations were found between LBP and psychological factors and between negative perception of the consequences of the disease and low-efficacy and depression. Wand et al.^25^ also highlighted that the greater the LBP-related disability was, the greater the likelihood of presence of symptoms of depression and vice versa^25^.

Based on the results of this study, health professionals should be alert during the screening and evaluation of elderly patients with acute LBP in order to identify disability conditions, symptoms of depression, and lower expectations regarding return to activities. These factors could be identified to avoid a chronic pain condition that might lead to long-term functional decline^7^. In light of this, healthcare professionals should identify the psychological “yellow flags” in patients with acute LBP^4^ and follow guidelines^7^, advising patients to continue their daily activities.

In addition to physical effects, LBP is associated with psychosocial outcomes^27^. The present study is relevant due to its analysis of attitudes and beliefs in a specific sample of elderly patients with acute LBP, which differs from samples of young patients, and it is part of an international consortium^10^ that has agreed to compare results in different cultures. It is also worth highlighting the sample size, which includes data from more than 500 elders.

One of the limitations of this study is that LBP is self-reported, however this type of assessment is common due to the subjectivity of LBP and it is well accepted in epidemiological studies^3^ ^,^ ^27^. Other limitations include the use of a convenience sample and the cross-sectional design, which did not provide information about cause-and-effect relationships. Future studies should verify this approach by investigating the attitudes and beliefs of elderly patients with acute LBP in a longitudinal analysis, as well as other samples with elderly patients with subacute and chronic LBP.

## Conclusion

Disability, symptoms of depression, and expectation of return to activities were associated with negative attitudes and beliefs concerning LBP in Brazilian elders with acute LBP. Screening for psychosocial factors and providing adequate recommendations to patients through preventive interventions might contribute to recovery from LBP and avoid the development of future disability.
